# The coagulation status in women of endometriosis with stage IV

**DOI:** 10.1186/s12905-024-03227-4

**Published:** 2024-07-04

**Authors:** Lu Wang, Jingxian Ling, Xianghong Zhu, Yan Zhang, Rong Li, Jingjing Huang, Doudou Huang, Chan Wu, Huaijun Zhou

**Affiliations:** Department of Obstetrics and Gynecology, Affiliated Hospital of Medical School, Nanjing Drum Tower Hospital, Nanjing University, Nanjing, China

**Keywords:** Endometriosis, Inflammation, Hypercoagulability, Stage IV

## Abstract

**Background:**

Endometriosis is considered as a systemic disease with the presence of proinflammatory cytokines in the circulation, which drives hypercoagulable state of endometriosis. Currently, endometriosis is classified into four stages: I (minimal), II (mild), III (moderate) and IV (severe). The aim of this study is to investigate the correlations between inflammatory markers and coagulation factors in patients diagnosed of endometriosis with stage IV.

**Methods:**

This retrospective case–control study included 171 endometriosis patients with stage IV and 184 controls. Continuous data were expressed by mean ± standard deviation. Mann–Whitney U and χ2 tests were used to compare the medians and frequencies among the groups. Spearman analysis was conducted to determine the correlation among the measured parameters. The diagnostic values of the parameters differentiating endometriomas were tested by receiver operating characteristic (ROC) curve.

**Results:**

The time of activated partial thromboplastin time (APTT) was decreased and the concentration of fibrinogen (FIB) and neutrophil-to-lymphocyte ratio (NLR) were increased in women of endometriosis with stage IV. The APTT were negatively correlated with NLR while the concentrations of FIB were positively correlated with NLR. The ROC analysis showed that the Area under the curve (AUC) of FIB was 0.766 (95% confidence interval:0.717–0.814) with sensitivity and specificity reaching 86.5 and 60.9%, respectively. The AUC of CA125 and CA199 was 0.638 (95% confidence interval: 0.578–0.697), 0.71 (95% confidence interval: 0.656–0.763) with sensitivity and specificity reaching 40.9 and 91.8%, 80.7 and 56.5% respectively. The combination of these factors showed the highest AUC of 0.895 (0.862–0.927) with sensitivity of 88.9% and specificity of 77.7%.

**Conclusion:**

In the present study, we found that inflammatory factors showed significant correlation with APTT or FIB in endometriosis with stage IV. Moreover, the coagulation factors combined with CA125 and CA199 were more reliable for identifying the endometriosis with stage IV.

**Supplementary Information:**

The online version contains supplementary material available at 10.1186/s12905-024-03227-4.

## Introduction

Endometriosis is a chronic disease characterized by the presence of endometrial-like tissue outside the uterus [1]. This disorder is estimated to affect 5–10% of women of reproductive age [2]. Endometriosis is observed in 40–50% of women with persistent pelvic pain and up to 50% of women with infertility [3]. The diagnosis of endometriosis is typically delayed by years although it is prevalent. The laparoscopy is considered as the gold standard of diagnosis for endometriosis. Heretofore, there is no reliable blood diagnostic biomarkers for endometriosis [4].

Currently, endometriosis is usually treated by surgery therapy. Endometriosis is recognized classified into four stages: I (minimal), II (mild), III (moderate) and IV (severe) [5]. Women of endometriosis with stage IV showed extensive lesions and more pelvic adhesions, which making surgery therapy more difficult [6]. It is necessary for us to identify the patients of endometriosis with stage IV before surgery, which could help us adequately prepare for surgery.

Endometriosis is a systemic inflammatory condition with the presence of proinflammatory cytokines in the circulation [7, 8]. In patients with endometriosis, the inflammation leads to the endometriosis-associated pain, endothelial dysfunction and carcinogenesis even though [7, 9]. The activation of inflammation also drives hypercoagulable state [10, 11]. The decreased levels of thrombin time (TT), activated partial thromboplastin time (APTT) and increased level of tissue factor (TF), fibrinogen have been found in patients with endometriosis [12–15]. However, the correlation between inflammatory markers and coagulation factors of endometriosis with stage IV has not been clearly clarified.

In the present study, we aimed to investigate the correlations between inflammatory markers and coagulation factors in patients diagnosed of endometriosis with stage IV. We also explored the diagnostic value of these parameters in endometriosis with stage IV, which provide new ideas for diagnosis with auxiliary biomarkers.

## Methods

### Patients

This research was approved by the ethics committee of the Nanjing Drum Tower Hospital (2024-094-01) in accordance with the Declaration of Helsiniki. A total of 355 patients including 171 endometriosis patients with stage IV and 184 controls who underwent surgery between January 2022 and December 2023 was participated. The stage of endometriosis was established according to the revised classification of the American Society for Reproductive Medicine [5]. The control group included patients with a surgical diagnosis of uterine leiomyomas, tubal pathology and ovarian benign cysts. All patients included in this research owned a histopathological diagnosis after either laparoscopic or laparotomic surgery. The patients in control group showed no stage of endometriosis by both the surgical and the histopathological examinations. All women have provided their informed consent for their clinical data to be used for researches purposes. The inclusion criteria were as follows: nonpregnant reproductive-age women and a surgical indication for endometriosis or other pelvic diseases. The exclusion criteria were as follows: patients in pregnancy or menopause; patients complicated autoimmune, hematological or blood coagulation diseases; patients with abnormal hepatic and renal function tests; patients who had taken corticosteroids and contraceptives within 3 months before surgery; patients with a diagnosis of malignancy.

### Blood assay

Before surgery, all patients had routine blood analyses including complete blood count parameters, serum biochemistry, activated partial thromboplastin time (APTT), thrombin time (TT), prothrombin time (PT), fibrinogen (FIB), neutrophil-to-lymphocyte ratio (NLR), serum carbohydrate antigen (CA) 125 and CA199. The NLR was obtained from dividing the absolute neutrophil count by the absolute lymphocyte count. All blood analyses were established during either the follicular or the luteal phase before surgery.

### Statistical analysis

The data was analyzed by SPSS 22.0 software (Chicago SPSS Co., Ltd.). Continuous data were expressed by mean ± standard deviation. Mann–Whitney U and χ2 tests were used to compare the medians and frequencies among the groups. Spearman analysis was conducted to determine the correlation among the measured parameters. The diagnostic values of the parameters differentiating endometriomas were tested by receiver operating characteristic (ROC) curve. *P* < 0.05 is considered as statistical difference.

## Results

### Patients’ characteristics

It was found that there were no statistical differences in age, BMI between Endometriosis with stage IV and Control groups (*P* > 0.05). Compared with the Control group, the parity was significantly lower, and the incidence of dysmenorrhea was significantly higher in the Endometriosis group (*P* < 0.05). No statistical difference was observed in the size of ovarian cysts between the Endometriosis and Control groups (*P* > 0.05, Table 1).

**Table 1:** Baseline characteristics of controls and patients of endometriosis with stage IV.

Characteristics	Controls	Endometriosis with stage IV	*P*.value
	*n* = 184	*n* = 171	
Age, years	31.27 ± 5.11	30.81 ± 5.07	0.40
BMI, kg/mq	24.20 ± 4.30	24.14 ± 4.73	0.89
Smoking, n (%)	16.85	24.56	0.07
Previous delivery, n (%)	14.13	11.11	0.39
Dysmenorrhea, n (%)	36.96	42.69	0.27
Cyst size, (cm)	6.45 ± 0.84	6.81 ± 0.73	0.08
Cycle phase: proliferative; secretory	48.37%;51.63%	48.53%;51.46%	0.98

BMI, body mass index

### The comparisons of inflammatory and coagulation markers between control and endometriosis with stage IV

**Fig. 1:**
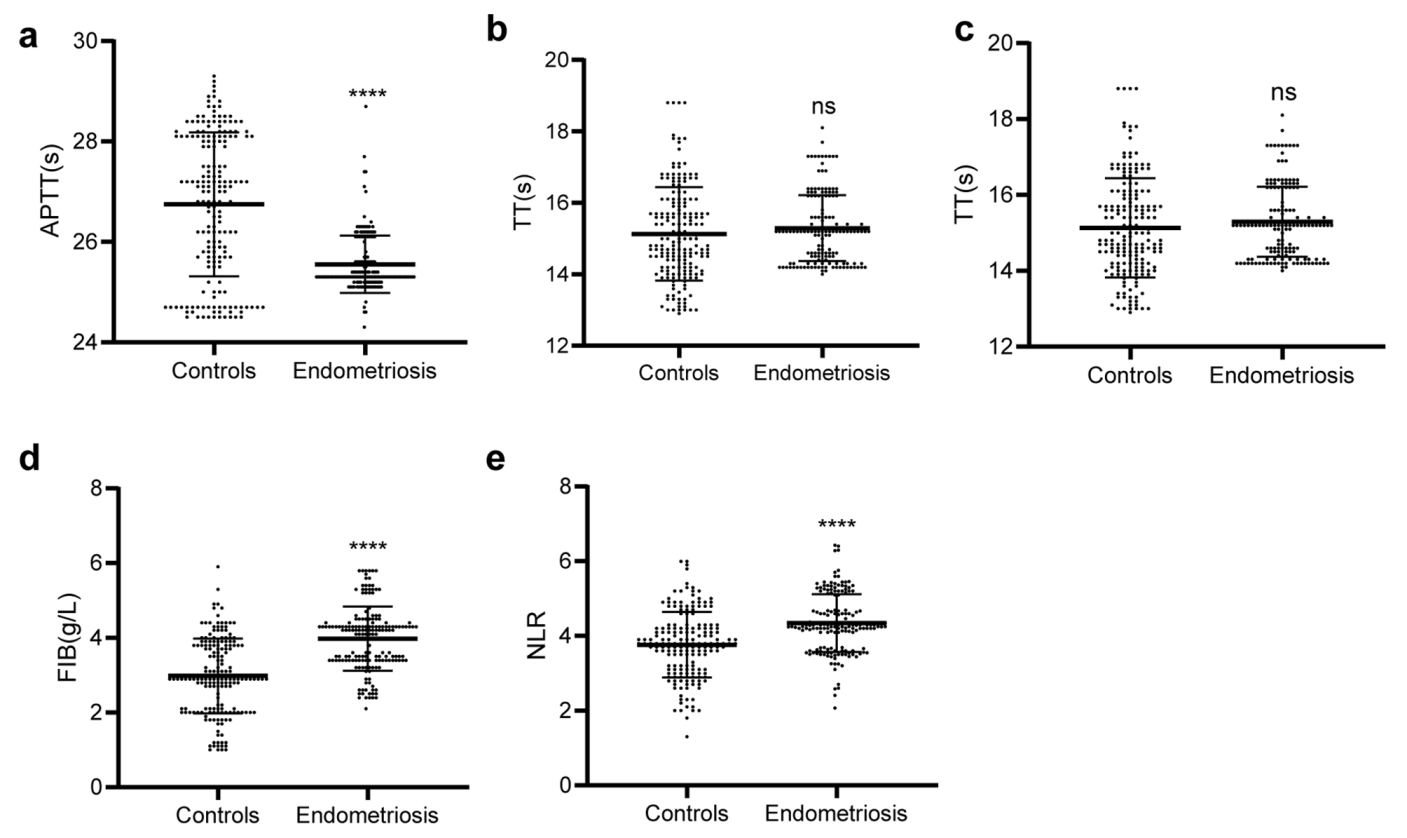
The coagulation factors and NLR in groups. **a-d**, Coagulation factors of APTT(a), TT(b), PT(c) and FIB(d) between controls and endometriosis with stage IV. e, NLR between controls and endometriosis with stage IV. APTT, activated partial thromboplastin time; TT, thrombin time; PT, prothrombin time; FIB, fibrinogen; NLR, neutrophil-to-lymphocyte ratio. ** *P* < 0.01, **** *P* < 0.0001

**Fig. 2:**
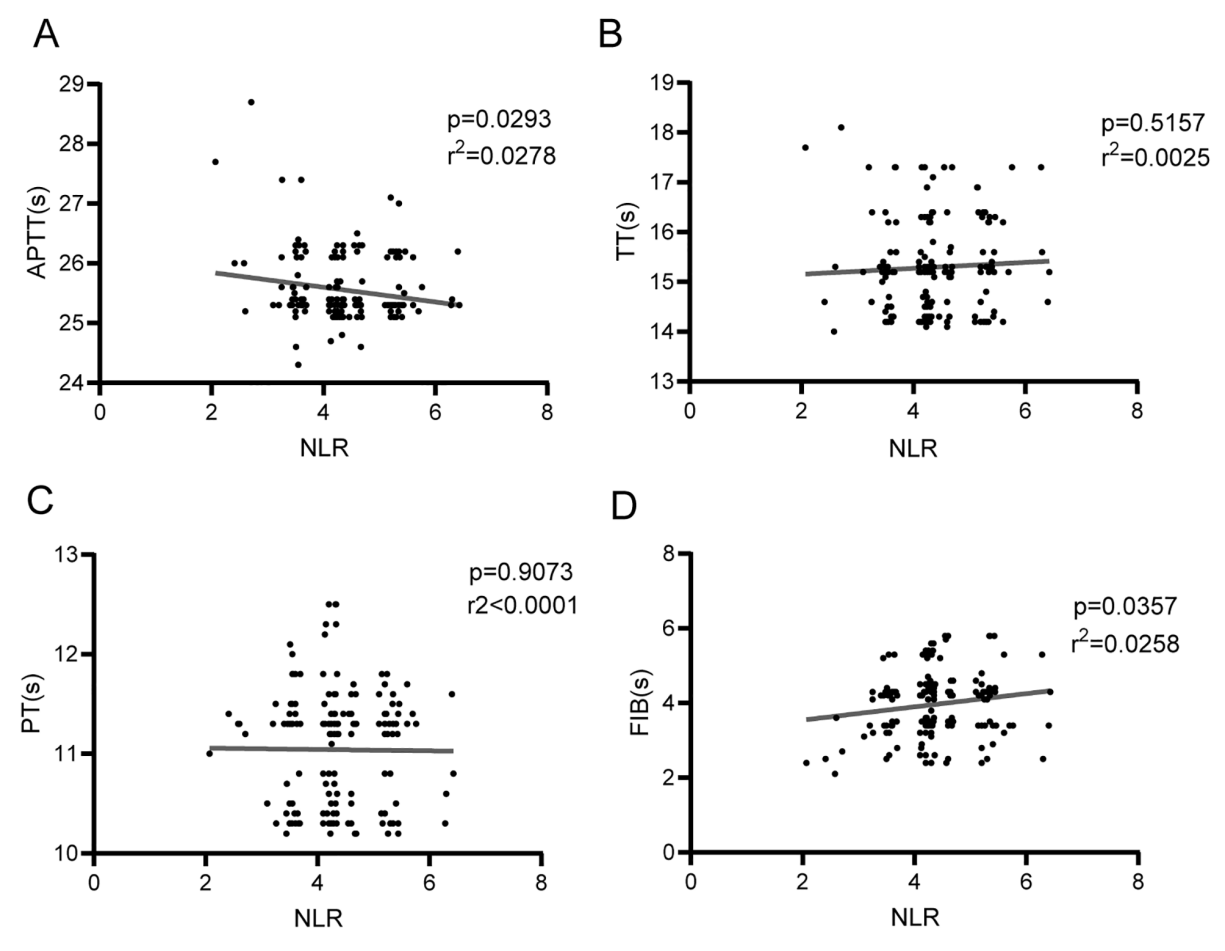
Correlations of coagulation factors and NLR in women of endometriosis with stage IV. **a-d**, The correlations of NLR with APTT (a), TT (b), PT (c) and FIB (d) were tested using Spearmen analysis. APTT, activated partial thromboplastin time; TT, thrombin time; PT, prothrombin time; FIB, fibrinogen; NLR, neutrophil-to-lymphocyte ratio

To explore the coagulation state in women of endometriosis with stage IV, we compared the APTT, TT, PT and FIB between two groups. As shown in Fig. 1a, the time of APTT was significantly decreased in women of endometriosis with stage IV. The concentration of FIB was apparently increased in women of endometriosis with stage IV (Fig. 1d). However, the time of TT and PT showed no difference between two groups (Fig. 1b-c). These results showed that the women of endometriosis with stage IV exhibited a high coagulation state. Meanwhile, we also compared the inflammatory state between two groups. The level of NLR increased in women of endometriosis with stage IV, which showed no significant difference between proliferative and secretory phase ((Fig. 1e and Supplementary Fig. 1). Besides, we investigated the correlation between coagulation factors and NLR. The correlation analysis showed that the APTT were negatively correlated with NLR while the concentrations of FIB were positively correlated with NLR (Fig. 2a-d). These results showed that the coagulation factors are closely related to inflammation.

### The diagnostic value of coagulation factors, CA125 and CA199 in endometriosis with stage IV

**Fig. 3:**
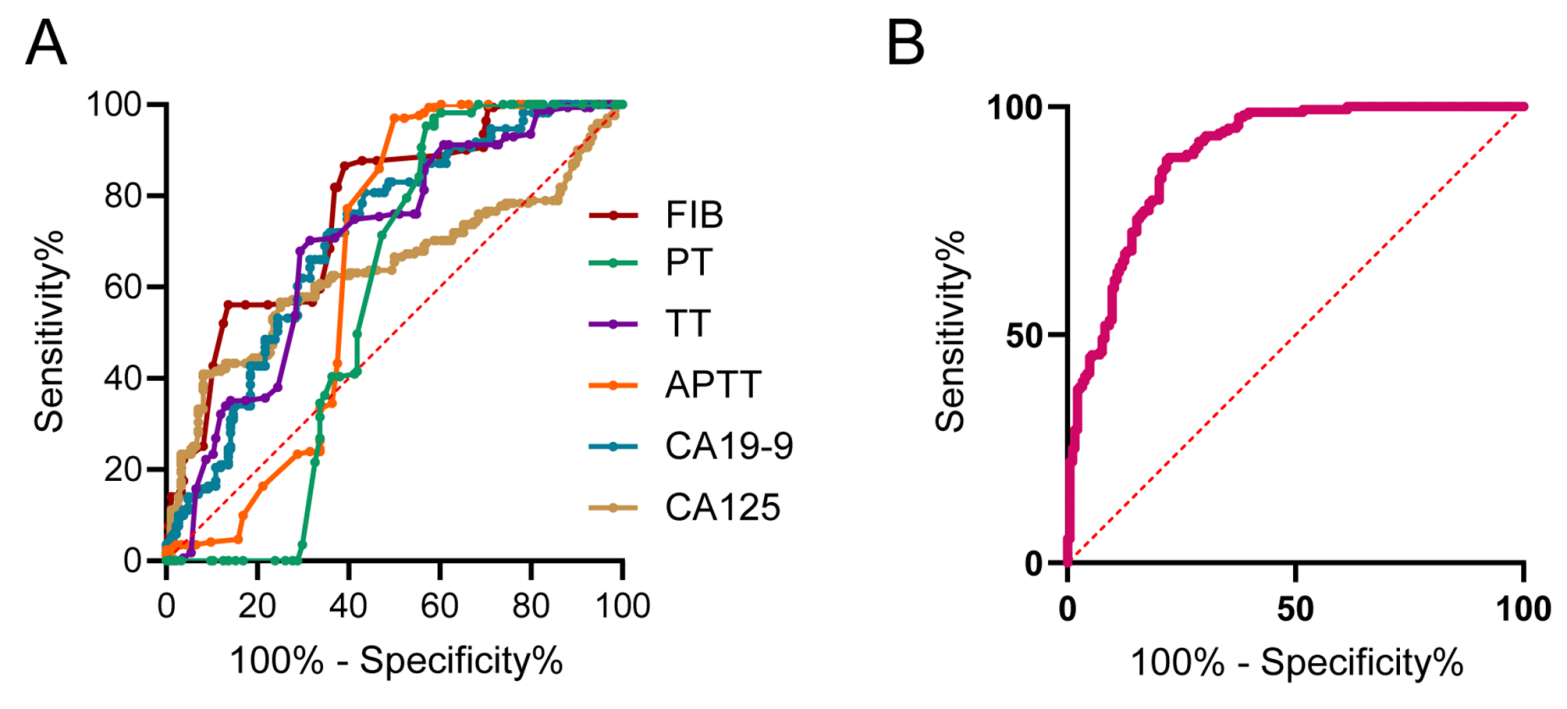
Receiver operating characteristic curves of CA125, CA199, coagulation factors and the combined marker for the diagnosis of endometriomas with stage IV. **a**, The ROC analysis of CA125, CA199 and coagulation factors for the diagnosis of endometriomas with stage IV. **b**, The ROC analysis of combined marker for the diagnosis of endometriomas with stage IV. ROC, receiver operating characteristic; APTT, activated partial thromboplastin time; TT, thrombin time; PT, prothrombin time; FIB, fibrinogen; NLR, neutrophil-to-lymphocyte ratio

We investigated the diagnostic effects of coagulation factors, CA125 and CA199 by ROC analysis. As shown in Table 2; Fig. 3a, among coagulation factors, FIB exhibited maximum Area under the curve (AUC) which was 0.766 (95% confidence interval:0.717–0.814) with sensitivity and specificity reaching 86.5% and 60.9%, respectively. The AUC of CA125 was 0.638 (95% confidence interval: 0.578–0.697) with sensitivity and specificity reaching 40.9% and 91.8%, respectively. Besides, the AUC of CA199 was 0.71 (95% confidence interval: 0.656–0.763) with sensitivity and specificity reaching 80.7% and 56.5%, respectively. However, the combination of these factors showed the highest AUC of 0.895 (0.862–0.927) with sensitivity of 88.9% and specificity of 77.7% (Table 2; Fig. 3b).

**Table 2:** The diagnostic value of coagulation factors, CA125, CA199 and combined factor in patients of endometriosis with stage IV.

parameters	AUC (95%CI)	Sensitvity(%)	Specificity(%)	cutoff value	*p*
APTT	0.651(0.590–0.713)	97.1	50	0.471	< 0.0001
TT	0.695(0.640–0.750)	70.2	68.5	0.387	< 0.0001
PT	0.576(0.512–0.641)	97.1	41.3	0.384	0.013
FIB	0.766(0.717–0.814)	86.5	60.9	0.474	< 0.0001
CA125	0.638(0.578–0.697)	40.9	91.8	0.327	< 0.0001
CA199	0.71(0.656–0.763)	80.7	56.5	0.372	< 0.0001
combined marker	0.895(0.862–0.927)	88.9	77.7	0.666	< 0.0001

APTT, activated partial thromboplastin time; TT, thrombin time; PT, prothrombin time; FIB, fibrinogen; CA125, serum cancer antigen 125; CA199, serum cancer antigen 199

## Discussion

In summary, we confirmed that the coagulation factors were changed in women of endometriosis with stage IV in this study. APTT and FIB showed significantly correlation with NLR. Moreover, the combination of coagulation factors with CA125 and CA199 showed useful diagnostic value for endometriosis with stage IV. However, we still have some limitations. Firstly, this research was a retrospective study. Secondly, the patients in controls were surgical population.

Endometriosis is a chronic and systemic disease which could cause persistent pelvic and infertility [16]. The symptoms involve deep dyspareunia, severe dysmenorrhea, as well as bladder and bowel fatigue [17]. This disorder is so complex and heretofore, there is no effective treatment. To some extent, hormonal and surgical treatments have limitations and they could not address the systemic syndrome. In addition, endometriosis is related to mental health, social life, affecting work productivity, which leads to a major economic burden [18–21]. Unfortunately, besides pathological diagnosis after laparoscopic examination, there is no other effective non-invasive diagnostic method.

Inflammation is an important response in the pathogenesis of endometriosis. Inflammation could lead to dysfunction of endothelial, even promotes carcinogenesis [7]. Many inflammatory factors, such as tumor necrosis factor (TNF)-alpha, interleukin (IL)-1beta, IL-33 and IL-6, were upregulated in peritoneal fluid of women with endometriosis [22, 23]. Besides, inflammatory cells, including neutrophils and macrophages, were activated in endometrium of women with endometriosis [24]. Moreover, increased evidences indicated that there existed a cross-talk between inflammation and coagulation, whereby the initiation of inflammation could lead to the activation of coagulation [12]. In the present study, we found that inflammatory factors showed significant correlation with APTT or FIB in endometriosis with stage IV.

The combination of CA125 and CA199 showed diagnostic value of the endometriosis [25, 26]. Nevertheless, the predictive value of individual markers was limited. Some studies reported that CA125 combined with NLR showed useful diagnostic values, but the combination was not suitable for advanced endometriosis [27–29]. In our study, we found that coagulation factors combined with CA125 and CA199 were more reliable for identifying the endometriosis with stage IV.

In conclusion, inflammation and coagulation factors are crucial in the pathogenesis of endometriosis. Further studies on coagulation parameters or inflammatory markers combined with tumor markers would help to identify a new therapeutic strategy.

### Electronic supplementary material

Below is the link to the electronic supplementary material.

Supplementary Material 1

## Data Availability

Data is provided within the manuscript or supplementary information files.
