# Supplementary material for: The coagulation status in women of endometriosis with stage IV
Source: BMC Womens Health. 2024 Jul 4;24:386. doi: 10.1186/s12905-024-03227-4 (PMC11223421; doi:10.1186/s12905-024-03227-4)
Supplement: Supplementary file 1 — Supplementary Material 1 [file 12905_2024_3227_MOESM1_ESM.docx]

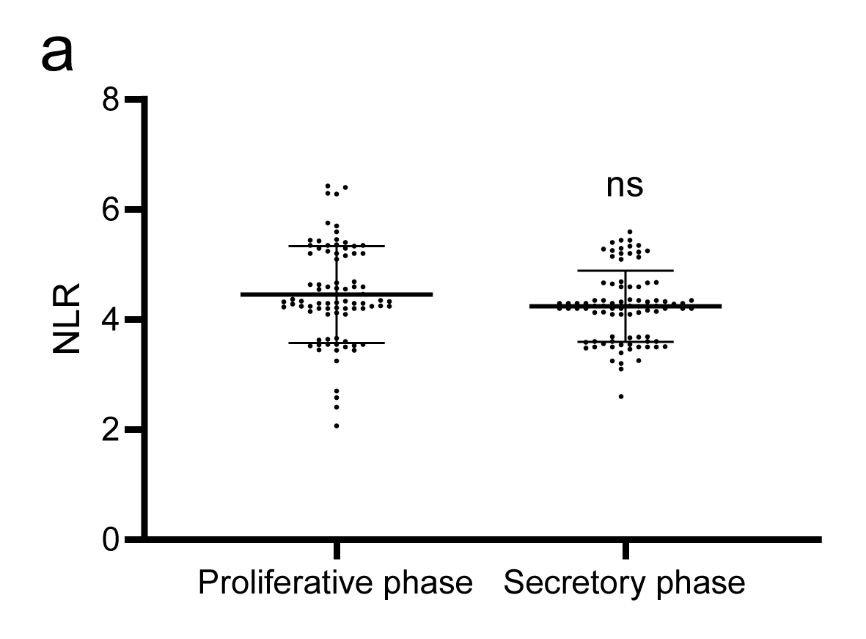


**Figure 1. The NLR** **in endometriosis with stage IV.**

**a**, NLR between proliferative and secretory phase in endometriosis with stage IV.

NLR, neutrophil-to-lymphocyte ratio. ns, no significance.
